# Variants in exons and in transcription factors affect gene expression *in trans*

**DOI:** 10.1186/gb-2013-14-7-r71

**Published:** 2013-07-11

**Authors:** Anat Kreimer, Itsik Pe'er

**Affiliations:** 1 Department of Biomedical Informatics, Columbia University, 622 West 168th Street, New York, NY 10032, USA; 2 Center of Computational Biology and Bioinformatics, Columbia University, New York, NY 10032, USA; 3 Department of Computer Science, Columbia University, 500 West 120th Street, New York, NY 10027, USA

**Keywords:** Computational biology, eSNPs, eQTLs, protein-protein-interaction networks, regulation, regulatory networks, systems biology, systems genetics, transcriptome

## Abstract

**Background:**

In recent years many genetic variants (eSNPs) have been reported as associated with expression of transcripts in *trans*. However, the causal variants and regulatory mechanisms through which they act remain mostly unknown. In this paper we follow two kinds of usual suspects: SNPs that alter coding regions or transcription factors, identifiable by sequencing data with transcriptional profiles in the same cohort. We show these interpretable genomic regions are enriched for eSNP association signals, thereby naturally defining source-target gene pairs. We map these pairs onto a protein-protein interaction (PPI) network and study their topological properties.

**Results:**

For exonic eSNP sources, we report source-target proximity and high target degree within the PPI network. These pairs are more likely to be co-expressed and the eSNPs tend to have a *cis *effect, modulating the expression of the source gene. In contrast, transcription factor source-target pairs are not observed to have such properties, but instead a transcription factor source tends to assemble into units of defined functional roles along with its gene targets, and to share with them the same functional cluster of the PPI network.

**Conclusions:**

Our results suggest two modes of *trans *regulation: transcription factor variation frequently acts via a modular regulation mechanism, with multiple targets that share a function with the transcription factor source. Notwithstanding, exon variation often acts by a local *cis *effect, delineating shorter paths of interacting proteins across functional clusters of the PPI network.

## Background

Creating the complete human regulatory map is an active field of study. Many previous studies have used genomic analyses of gene expression, binding motifs, epigenetic marks and other local features to infer regulatory interactions [1-5]. In recent years it has been established that genetic variation can contribute an additional angle to this investigation [6-9]. Formally, transcription level is considered as a quantitative trait that is altered by allelic variation with thousands of single nucleotide polymorphism (SNPs) reported as associated with changes in gene expression [10-13]. Such markers, called expression SNPs (eSNPs) are further found to contribute to variation of disease phenotypes and other clinically relevant traits [14-16].

Variation in genomic DNA can affect transcription in multiple ways. Most intuitively perhaps, level of transcripts *in cis *of an eSNP may be altered due to allelic variation in regulatory elements [17]. Alternatively, such levels may be auto-regulated by changes in protein structure that reflect variation of the sequence content of local transcripts. Therefore, *cis *eSNPs have been studied extensively. However, *cis *associations are limited in their ability to inform us regarding the network of regulatory interactions between one gene and another. This motivates more focused study of the effects of genetic variants on expression of distal transcripts (*trans *associations). Unfortunately, while *trans *eSNPs can identify downstream effects and previously un-annotated regulatory pathways, they are harder to statistically and biologically justify than *cis *eSNPs. From a statistical perspective, since *trans *eSNPs can be associated with any distal transcript, the multiple testing burden dramatically increases, thus only a small number of results is detected. From a biological perspective, more complex mechanisms are needed to explain *trans *associations. An example of such a mechanism is an eSNP with local *cis *effect on a gene which codes for a transcription factor known to regulate other genes *in trans*. Indeed, across multiple eSNP studies [7,18], even when statistically significant *trans *or *cis *eSNPs associations are detected aplenty, the regulatory mechanisms by which they alter gene expression remain mostly unknown.

A large fraction of SNPs identified by genome-wide association studies (GWAS) [10] have been reported to be associated with disease phenotypes [14] despite being neither coding, nor linked to coding SNPs *in cis*. Furthermore, since large-scale genetic studies have been predominantly based on SNP arrays, SNP alleles that are reported as associated, in studies of either disease [10] or gene expression [7], are often merely tags for causal variants, whose identity is challenging to track down. More generally, the multitude of phenotypes for eSNPs represents an opportunity for tackling the central question of causation in association.

Protein-protein interaction (PPI) networks capture various experimental data, such as from yeast two-hybrid systems [19], regarding the physical binding of proteins, and are often used to examine how these interactions are involved in a specific biological function. Recently, improved data on signal transduction and metabolic and molecular networks have contributed to the fidelity and accuracy of the reconstructed PPI networks. However, the data represented by these networks can sometimes be partial and noisy. PPI networks have been modeled as theoretical graphs and their topological properties extensively studied [20-22]. This provided insights pertaining to functional, structural and evolutionary characterization of these networks, primarily in model organisms. Genetic interactions in yeast were studied in the context of protein complexes network [23], motivating the investigation of genetic variants that alter gene expression (as interactions) with respect to the human PPI network[24]. Studies of PPI networks in the context of genetic variation have thus far focused on GWAS-detected SNPs that are associated with common traits and disease, reporting that genes that harbor such SNPs frequently code for interacting proteins [24-28]. Yet, such studies only considered the PPI-network nodes that correspond to the associated SNP, without a PPI network node that would correspond to the phenotype.

Here, we perform a comprehensive study of *trans *genetic associations and their large-scale properties as manifested on a PPI network. We use SNPs from sequencing data [29] that are candidates to be causal based on their genomic location, and then project their association to gene expression on a PPI network. We hypothesized that genes involved in true eSNP associations have distinct PPI-network properties that differ significantly from spurious genes with candidate association signals. To address this hypothesis, we focus on *trans *association of eSNPs in exons and transcription factors (TFs), analyzing their properties as reflected on the PPI-network topology and annotations of the genes involved. Our focus on expression quantitative traits allows consideration of paths along the PPI network, whose links with genetic variation had previously only been studied with respect to SNPs, rather than the transcripts they modulate.

Our results suggest that a significant fraction of eSNPs in exons act *in trans *through mild effects *in cis*, with a regulation mechanism that is mediated by PPI paths that are shorter than expected by chance and tend to traverse across functional clusters of the PPI network. These paths highlight zinc ion binding genes as a possible mechanism of transcript-eSNP feedback across the PPI network. In comparison to such coding eSNPs, we observe that TFs harboring eSNPs and their associated genes create units of genes that are functionally enriched for biological annotations. This suggests a different, modular regulatory mechanism for such TF eSNPs. Altogether, our analysis offers insights concerning a variety of mechanisms by which genetic variation at functional loci shapes the structure of human regulatory networks.

## Results

### Computational framework for mapping trans associations onto the PPI network

We were interested in pinpointing directly associated variants rather than indirectly imputed ones. We thus used a publicly available dataset of 50 fully sequenced Yoruban samples [29] along with their transcription profiles from RNA-sequencing data [30], bearing in mind that such available cohorts are limited in size. Due to this small sample size, we have limited power in detecting association. Therefore, most candidate eSNPs can only be designated with various levels of uncertainty.

We were intrigued to examine *trans*-eSNPs interactions with respect to an independent space of interactions, that is, a PPI network. Therefore, we evaluated two categories of candidate eSNPs that reside within regions along the genome with known regulatory potential and can be mapped onto a PPI network, that is, exons and TFs (Figure S1 and Table S1 in Additional file 1; see Methods). Examining the distribution of *P*-values across these two categories of candidate *trans*-eSNPs, we observed that candidate eSNPs within exons show evidence of including true positive eSNPs (Figure S2a in Additional file 1), as been previously shown [31]. By contrast, eSNP candidates in TFs show association signal distributions consistent with random expectation (Figure S2b in Additional file 1). We further examine if TF candidate eSNPs exhibit qualities that are different from random. We hypothesized that a single TF will be associated with multiple transcripts via eSNPs. To address this hypothesis, we created 1,000 permuted sets of pairs of TF and transcript (see Methods). We observed that the number of multiple associated transcripts is significantly higher (Wilcoxon rank sum test *P *<0.05) in the real dataset (973 out of 1,000 permuted sets, empirical *P*-value = 0.027). Following these two observations, we focused on eSNPs within exons as the first subject of our investigation, and compared them to eSNPs within the span of transcription factor genes. We set out to characterize and compare these two modes of *trans *regulation.

**Figure 1:**
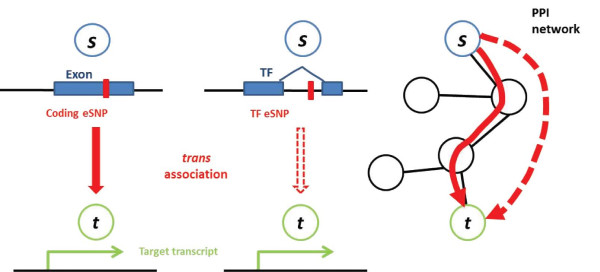
***trans *associations (solid and dashed red straight arrows) on a protein-protein interaction network**. An eSNP (red tick mark) that resides within a known exon (left) or TF (middle) maps to the PPI network (right). The source gene (blue *s*) is associated *in trans *with the levels of a target transcript (green *t*). PPI network edges are denoted in black, and define the shortest path between the exon source and its target (solid red curved arrow). The association between an eSNP within a TF source and its gene target is denoted by a dashed red curved arrow. eSNP, expression single nucleotide polymorphism; PPI, protein-protein interaction; TF transcription factor.

For each candidate eSNP that is associated with levels of a transcript in *trans*, we denoted this transcript as the 'target' of the eSNP. When this eSNP was located within an exon or in the span of a TF, we defined this gene as 'source'. We attempted to characterize eSNPs interactions on the molecular level by mapping these pairs of source-target genes onto a PPI network (see Methods and Figure 1) and studied their functional annotations and topological properties.

### Identifying topological properties of exonic eSNP interactions

We first considered pairs of exon eSNP source and target that demonstrated an association signal which was significant exome-wide for a particular transcript (association *P *<10^-7^). We observed such pairs to be significantly closer (*P *= 0.03) on the PPI network when compared with randomly permuted candidate eSNPs (see Methods). Beyond pairwise properties of sources and targets, we further attempted to characterize each by their single-node features. Specifically, the targets of exon eSNPs had significantly higher (*P *= 0.003) degree than expected based on random pairs.

**Figure 2:**
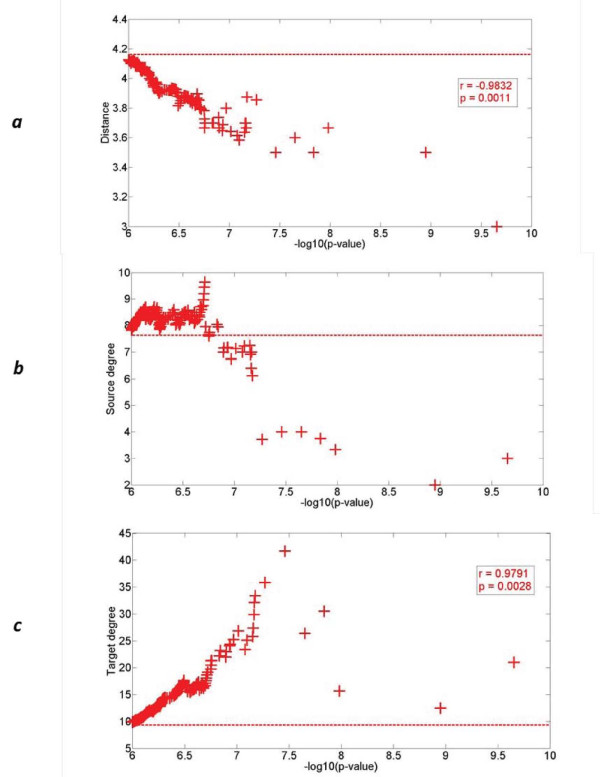
**Topological properties on a protein-protein interaction network versus exonic source-target association significance**. Averages for **(a) **distance between source and target, **(b) **source degree and **(c) **target degree are evaluated across source-target pairs of candidate exon eSNPs at varying association p-value thresholds (+). The average of randomly permuted pairs (dashed horizontal line) is shown for permuted pairs and Spearman's rank correlation coefficient (denoted r) is listed when significant at *P *<0.05 (denoted p).

We reasoned that the cutoff of association *P*-value we used (*P *<10^-7^) was in many ways arbitrary, as we were interested in the statistical properties of the set of results rather than the significance of a particular result amid the testing burden. We therefore considered multiple *P*-value thresholds of eSNP association, and at each threshold evaluated topological properties of eSNP source and target pairs, while assessing significance vis-à-vis randomly permuted sets of candidate eSNPs in exons (see Methods). We observed that the lower the association *P*-values for source-target pairs, the more their topological properties differed compared with random pairs (Table S2 in Additional file 2). For example, for source-target pairs of exon eSNP, the average target degree among the 52 pairs exceeding an association *P*-value cutoff of 10^-6.5 ^was 16.42, but it reached as much as 22.22 among the more focused set of 22 pairs that exceeded association *P*-value cutoff 10^-6.8^. These averages were each significant (*P *= 0.02 and 0.006, respectively) when compared with permuted pairs of exon eSNPs, whose target degree was only 9.36 on average. These trends are consistent with properties of true positives being diluted by false positives at less significant *P*-value thresholds. We quantified such trends by regressing each topological property on the negative log10 of the association *P*-value (Figure 2). We confirmed that for exonic source-target pairs, network distance decreased and the target degree increased with the significance of association (Spearman rank correlation coefficients r = -0.98 and 0.97, respectively; permutation *P*-value *P *= 0.001 and 0.002, respectively - see Methods).

These results highlight unique properties of part of the transcripts whose *trans *regulation is due to coding variation. Specifically, we show that loci implicated by eSNPs encode for proteins that physically interact in a non-random fashion. Furthermore, target proteins are likely to interact with significantly more nodes of the PPI network than expected by chance.

### Characterization of exon and transcription factor sources and targets

Based on these results, for further analysis, we focused on the maximal *P*-value cutoff of 10^-6.463^, for which all topological properties showed significant difference between true source-target pairs of exon eSNPs and random ones (Wilcoxon rank sum test *P *<0.05), (Figure S3 and Table S3 in Additional file 1s Table S2 in Additional file 2).

There were 343 pairs of source and target and 295 unique pairs, 59 of them on the network. Of these pairs, 318 (92.71%) were on different chromosomes and 25 (7.29%) were on the same chromosome, at least 1 Mb apart (See Table S4a in Additional file 1). At this cutoff there were 333 unique eSNPs in exons, 286 unique gene sources and 267 unique gene targets (Table S5 in Additional file 3). When comparing the effect sizes (absolute values of betas in the linear regression) of 929 previously published *cis *expression quantitative trait loci (eQTLs) [30] with the distribution of exonic and TF *trans *eSNPs effect sizes, we found that the *trans *effect sizes (mean 1.198) were significantly higher than those of corresponding *cis *effects (mean 0.964; Wilcoxon rank sum test *P*-value <2.25 × 10^-49 ^and 3.56 × 10^-54 ^for exonic and TF eSNPs, respectively; Figure S4 in Additional file 1). We binned eSNPs and SNPs in exons by first, middle and last exons (See Figure S5 in Additional file 1). We also examined the position of the eSNP along the transcript and compared these results to SNPs in exons (See Figure S6 in Additional file 1). We observed that these *trans *exonic eSNPs tended to be located along middle exons, rather than in first or last exons (Fisher's exact test *P*-value <0.009). We further observed that they tended to lie farther away down the transcript (Wilcoxon rank sum test *P *= 0.0058). These results were different from what was observed for *cis *eQTLs. Montgomery *et al. *[32] reported that eQTLs with higher confidence were located in the first and last exons significantly more than in middle exons.

The combined set of exon sources was enriched for major histocompatibility complex protein genes (false discovery rate (FDR) <0.046) with concordance to findings in previous studies, indicating human leukocyte antigen SNPs were 10-fold enriched for *trans*-eSNPs [33]. We further observed that the set of target genes was enriched for multitude functional processes (see Table S6 in Additional file 4 for full list of annotations). The three highest scoring functional annotations of the target set, macromolecule modification, phosphatidylinositol-3,5-bisphosphate binding and protein modification process, provide additional support for the role of exonic eSNP targets as network hubs [34].

For further investigation and comparison, we considered source-target pairs of TF candidate eSNPs, a set with similar order of magnitude, corresponding to association signals passing the *P*-value cutoff of 10^-6^. There were 370 such pairs of TF source-target, 193 of them unique, 58 of which were on the network. Of these pairs, 359 (97.03%) were on different chromosomes and 11 (2.97%) were on the same chromosome, at least 1 Mb apart (See Table S4b in Additional file 1). There were 358 unique eSNPs in TFs, 77 unique TF sources and 192 unique targets (Table S5 in Additional file 3). Out of the 358 unique eSNPs in TFs, 15 were in exons, significantly more than expected by chance (hypergeometric *P-*value <1.8×10^-4^). When we examined the combined set of TF targets, we observed that this gene set was enriched for various annotation categories (see Table S6 in Additional file 4 for full list of annotations).

To further establish the association between the source and target genes, we examined the co-expression between eSNP source and target for all candidate pairs of associated genes in this dataset by evaluating Spearman's rank-correlation coefficient *r*. For pairs of exon-source eSNPs and their corresponding targets, the absolute value of *r *was significantly higher than expected from the entire distribution of co-expression measurements in this dataset (Wilcoxon rank sum test *P *<5.4×10^-5^). By contrast, for pairs of TF-source eSNPs and their corresponding targets, there was no significant difference in terms of co-expression. We observed the fraction of non-synonymous SNPs to be 0.082 out of exon eSNPs, which was higher than their overall fraction 0.071 among all exonic SNPs [35] (Fisher exact *P *approximately 0.1). For each eSNP we examined *cis *effects that were too mild to be detected at genome-wide significance threshold by testing for its association with the expression of its source gene. In total, 50 pairs of exonic eSNP and source gene were nominally (*P *<0.05) *cis *associated, out of 286 such unique sources (*P *= 3.6 × 10^-15^). We estimated how many of the SNPs in exons have a *cis*-effect (linear regression *P*-value <0.05) on the expression of their host gene. We found that out of 97,135 exonic SNPs, 9,661 showed *cis*-effect on their host gene at the nominal significance level (*P *<0.05). Compared to this background distribution, the observed 50 out of 286 *trans *eSNPs having such *cis*-effects is significantly more than expected by chance (Fisher's exact test *P*-value <9.6 × 10^-5^). This provides additional support for the *cis*-effect phenomena. For comparison, we did not observe a nominally significant *cis *effect between TF eSNP and its source gene more than expected by chance (3 out of the 66 TF sources in this dataset). These results suggest a mechanism where exonic variation often operates in *trans *eSNPs via alteration of gene expression in *cis*, and the source and target genes have correlated expression.

TFs are known to control the transcription of multiple genes; we were therefore interested in whether we observed the same phenomena in TF variation. Each TF source forms, along with its targets, a set of genes that we called a unit. We observed that these units tended to be enriched for functional annotation categories. Specifically, for the 33 TF sources with two target genes or more (Tables S7 and S8 in Additional file 1), 26 out of 33 define units that are functionally enriched (two or more annotated genes, FDR <0.05) [36] in KEGG [37] and GO [38] categories (Table S9 in Additional file 5). Interestingly, eSNP targets did not tend to share exon sources. Specifically, out of 286 unique sources, 278 had a single target, 7 (*AKNA*, *CDK7*, *BLK*, *ATP5G1*, *RPL8*, *TRAPPC12*, *MUC2*) of the remaining ones had two, and one (*HLA-C*) had three (Table S5 in Additional file 3). The difference between the number of associated targets in TF and exon variation was statistically significant (Wilcoxon rank sum test *P *<3.4 × 10^-4^). These results support the hypothesis that TF variation frequently acts via a modular regulation mechanism, with multiple targets that share a function with the TF source.

We systematically looked for pairs of TF source-target that were experimentally validated as binding. We found such enrichment, with 6 out of 34 TF source-target pairs compared to 551 out of 6,904 random pairs (Fisher's exact test *P *<0.05, see Methods) in a database reporting binding of TFs to DNA, based on chromatin immunoprecipitation (ChIP)-X experiments [39]. We used the data in [40] to find the closest DNaseI hypersensitive site (DHS) window to the gene target, and examined whether the TF eSNP was associated with the DHS levels in this window. We found that 33 of 370 such pairs of TF eSNP and gene target were significantly associated (*P *<0.05) indicating significant enrichment (*P *<5.5 × 10^-4^) of this phenomenon. This enrichment was not an artifact of TF eSNP ascertainment: we tested the association of 29,212 TF SNPs to DHS levels in a randomly picked DHS window; as expected by chance, 1,400 of these SNPs showed such association at the nominal significance level, *P *<0.05. Compared to this background distribution, the observed set of 33 out of 370 *trans *eSNPs having such association was significantly larger than expected by chance (Fisher's exact test *P*-value <6 × 10^-4^). This shows that even in a small sample size where the number of true positives is diluted with false positives, we still recover a true signal.

We were intrigued by potential connections between source-target pairs and cluster properties in the PPI network. Therefore, we partitioned the PPI network into clusters of genes, optimizing the modularity measure [41]. Out of the resulting 249 PPI clusters with two genes or more, 225 (90%) demonstrated functional enrichment for a biological category (Table S10 in Additional file 6). TF source-target pairs were found in the same PPI clusters more than expected by chance: 26 out of 58 TF pairs compared with 26,966 out of 100,000 random pairs (Fisher's exact test *P *<0.0043).

**Figure 3:**
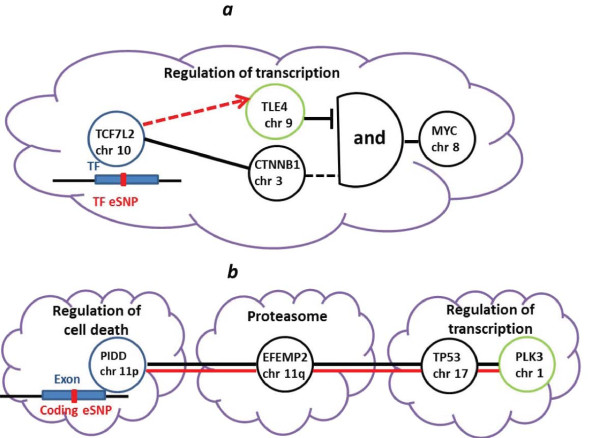
**Examples of transcription factors and exon source-target pairs**. An eSNP (red tick mark) along a source gene (blue circle), either in an exon or TF (blue rectangle), is associated (solid red line for exon, dashed for TF) with levels of transcription of the target gene (green circle). The source and target genes interact via nodes (black circles) and edges (black solid lines) in the PPI network. Each node belongs to a PPI cluster (purple cloud) with a functional annotation. **(a) **Network motif I1-FFL [42]: *TCF7L2 *activates *MYC *(in the presence of *CTNNB1*) but also represses *MYC *by activating the repressor *TLE4 *(via an eSNP) **. (b) **The shortest path on the PPI network between *PIDD *source and its gene target *PLK3*. Binding sites of *TP53 *were found in the promoter of *PLK3*. *TP53 *is annotated as a zinc ion binding protein. There was a significant correlation between the expression of the source and target genes. *TCF7L2*, transcription factor 7-like 2; T-cell specific; *TLE4 *transducin-like enhancer of split 4; *MYC*, v-myc avian myelocytomatosis viral oncogene; catenin (cadherin-associated protein), beta 1, 88kDa (*CTNNB1*); *PIDD*, p53-induced death domain protein; *PLK3*, polo-like kinase 3; *EFEMP2*, Epidermal growth factor-containing fibulin-like extracellular matrix protein 2; *TP53*, tumor protein p53.

As an illustration for our results, we show an example (Figure 3a) of a specific source and its gene target, examining transcription factor 7-like 2; T-cell specific, HMG-box (*TCF7L2*) and its transcript target transducin-like enhancer of split 4 (*TLE4*). There was a significant *cis *effect (*P *<0.012) of the associated intronic eSNP rs7087006 with the expression of *TCF7L2*, but the co-expression correlation of the source and target was not statistically significant in this dataset. *TCF7L2 *and its five targets (unit number 28, Table S8 in Additional file 1) comprise a unit that was enriched (two out of six) for cell proliferation (FDR <0.03; Table S9 in Additional file 5). This TF plays a key role in the Wnt signaling pathway, activating v-myc avian myelocytomatosis viral oncogene homolog (*MYC*) expression in the presence of catenin (cadherin-associated protein), beta 1, 88kDa (*CTNNB1*). The gene target *TLE4 *within the PPI network is a transcriptional co-repressor that represses transactivation mediated by *TCF7L2 *and *CTNNB1*. These annotations implicate that *TCF7L2*, *TLE4 *and *MYC *act as the network motif incoherent type-1-feed-forward loop (a pulse generator and response accelerator) [42] where the two arms of the feed-forward loop act in opposition: *TCF7L2 *activates *MYC *(in the presence of *CTNNB1*) but also represses *MYC *by activating the repressor *TLE4 *(via an eSNP). We note that *TCF7L2 *harbors the common allele most strongly associated with increased risk of type 2 diabetes. Correspondingly, *TLE4 *was recently discovered as a T2D locus [43]. Specifically, *TLE4 *encodes a protein that forms complexes with *TCF *proteins, including *TCF7L2*, to modulate transcription at target sites [44]. The source and target are part of the same PPI network cluster, which is enriched (1,257 out of 4,627) for regulation of transcription (FDR <2.4 × 10^-88^, Table S10 in Additional file 6; Figure 3a). This demonstrates a case of shared function between a source TF and its target.

By contrast, only 19 (32%) of exon eSNP sources were found in the same PPI network cluster as their respective single targets, consistent with chance expectation (Methods). Yet, as such pairs were linked by relatively shorter paths (Figure 2a), it follows that coding variants affect transcription *in trans *not in a modular way but rather in a linear fashion that defines shorter paths than expected by chance. We recorded the proteins along such paths (Table S11 in Additional file 1) and evaluated the enrichment of functional annotation for each path (Table S12 in Additional file 7).

We show an example (Figure 3b) of exon source and its gene target, examining the path between gene source p53-induced death domain protein (*PIDD*) and gene target polo-like kinase 3 (*PLK3*); path number 18, Tables S11 in Additional file 1 and S12 in Additional file 7). This path was enriched for the p53 signaling pathway (FDR <0.01, Table S12 in Additional file 7). *PIDD *promotes apoptosis downstream of the tumor suppressor as a component of the DNA damage/stress response pathway that connects p53 to apoptosis. The gene target *PLK3 *is a serine/threonine kinase that plays a role in regulation of cell cycle progression and potentially in tumorgenesis. Epidermal growth factor-containing fibulin-like extracellular matrix protein 2 (*EFEMP2*)and tumor protein p53 (*TP53*) reside along the shortest path between *PIDD *and *PLK3 *(Figure 3b). There is evidence from ChIP-ChIP and ChIP-seq experiments that *TP53 *has binding sites in the promoter of *PLK3 *[39] and it is annotated as a zinc ion binding protein. Furthermore, the combination of a pair of genes with TF-DNA and PPI edge between them is a known network motif (mixed-feedback loop) [45], suggesting a mechanism by which the expression of the target gene is altered. In support of this, the co-expression correlation of the source and target genes was significant (Spearman rank-correlation test *r *= 0.3223, *P *<0.02). The exon gene source and target reside in different PPI network clusters: *PIDD *resides in a cluster that is enriched for regulation of cell death (FDR <4.5 × 10^-6^, Table S10 in Additional file 6) and *PLK3 *resides in a cluster that is enriched for regulation of transcription (FDR <2.4 × 10^-88^, Table S10 in Additional file 6).

These results beg a mechanistic explanation that would clarify how the network interaction at the protein level is leading to the observed changes in transcript levels. Fortunately, examination of the genes along the reported paths provides a plausible answer, as they are strongly enriched for zinc ion binding proteins. Specifically, when we examined the enrichment for annotations of genes along shortest paths in the real dataset, we observed 410 enriched categories (minimum of 10 genes from a category, FDR <0.05; Table S13 in Additional file 8). For comparison, across 1,000 permuted datasets we observed a total of 1,870 categories satisfying the same enrichment criteria. We focus on the six categories that were enriched in real data and not in permutations: ion binding, metal ion binding, cation binding and intracellular, zinc ion binding and transition metal ion binding (Table S13 in Additional file 8). We compared two properties in real versus permuted datasets: first, the number of genes from each category (empirical *P*-values 0.005 and 0.014 for zinc ion binding and transition metal ion binding respectively); and second the number of paths where we observed at least one gene from each category (empirical *P*-values 0.016 and 0.038 for zinc ion binding and transition metal ion binding respectively). These results were replicated in a second permuted dataset. For comparison, only 7 and 10 out of the 404 joint categories achieve an empirical *P*-value lower than 0.05 for these two properties respectively. These results indicate that the genes in real paths were enriched for zinc ion binding, which is associated with regulation of transcription, suggesting a possible mechanism by which the expression level of the target transcript is modified.

## Discussion

We present a computational approach to study the characteristics of *trans *regulation. We observed that candidate eSNPs within exons exhibited an overabundance of significant association signals. We consequently focused on eSNPs that resided within an exon of a source gene, and were associated with the expression level of a different gene target. We observed that candidate eSNPs within TFs were associated with a higher number of transcripts than expected by chance. We subsequently examined eSNPs that resided within the span of source TFs. We mapped these pairs of source and target onto a PPI network and analyzed their topological properties.

We applied our approach to publicly available genetics and genomics [30] data from the same samples. We demonstrated that, by combining association data with information on PPI, it is possible to unravel topological properties for the two *trans *association types. We found that for an eSNP exon source and its gene target, the stronger the association, the closer the source-target distance and the higher the target degree in the PPI network. Expression analysis showed these source-target pairs to be frequently co-expressed, and that these exon eSNPs often had significant *cis *effects on the expression of the source genes. The observed phenomenon of exonic variation leaving a signature on PPI paths raises speculations regarding the mechanisms of transcription regulation. Previous studies have indirectly tackled these speculations regarding the connection between eSNP regulation and the PPI space. Specifically, Rossin *et al*. found that PPI connections between loci defined in GWAS of a specific disease were more densely connected than chance expectation [24], and Nicolae *et al. *[14] observed that SNPs found in GWAS were more likely to be eSNPs. The comprehensiveness of our work relied on combining eQTL data with the PPI network and not merely GWAS data, as described in previous studies [27]. This allowed us to examine source-target connections across the network, rather than be limited to studying the source nodes as in GWAS-PPI analyses. The novel observation is that the genetic variation that modifies PPI network properties is associated with a normal expression landscape and not only with extreme cases of disease.

We attempted to go beyond topological results and shed light on the regulatory mechanism by which gene expression of the target gene is altered in these shorter paths. We systematically compared genes along real and permuted shortest paths and found enrichment for ion zinc binding proteins, suggesting a plausible mechanism by which the expression level of the target transcript is modified. More generally, the paths of interacting protein pairs, from a source protein to the target protein, were consistent with concatenation of two pathways (Figure S7 in Additional file 1). The prefix of the path was consistent with a regulatory pathway, leading to some regulatory protein (TF or other) that affects expression of the target. The suffix of the path may match a self feedback loop in reverse: from the target protein back to the same regulatory protein [46].

We demonstrated it is possible to characterize regulatory variation in TFs. We observed that eSNP TF sources and their gene targets create units of genes that are enriched for functional annotations. When decomposing the PPI network to clusters, we observed that these source-target pairs tend to reside within the same cluster.

The design choices for a study of this kind convey a few methodological limitations. First, because we were interested in detecting putatively causal variants based on their exact genomic location, we used a dataset of fully sequenced individuals along with their transcription profiles. Such cohort sizes are limited in size, reducing the power to detect association and allowing us to see only the strongest effects. Second, we were interested in understanding the mechanisms underlying eSNPs interactions. This required the use of a well-established interaction network. We examined our results on a PPI network, rather than a TF-DNA interaction network or co-expression network derived from this dataset, to establish a broad and independent network of interactions. Overall, both the raw datasets [29,30] and supporting databases [37-39,47-49] in this work were noisy and limited. That we observed statistically significantly plausible results in such a small dataset combined with noisy databases is encouraging. Potentially, an increase in sample size may enable detection of eSNP associations at more significant *P*-values for even milder effects.

## Conclusions

Over the last decade, causal interpretation of genetic association signals for common variants and common traits had been impeded by two hurdles. First, many of the signals had been obtained as indirect association to proxy genetic markers, without access to the directly and causally associated variant. Second, often the trait under investigation was not understood at the molecular mechanistic level well enough to decipher the connection between variant and phenotype. This work bridges the gap between association and causality by considering both direct association to sequencing-ascertained variants, as well as expression quantitative traits. The ability to tie together these loose ends of genetic association using an interaction map constitutes a notable stride towards understanding the thousands of such connections that recent genetics have discovered.

**Figure 4:**
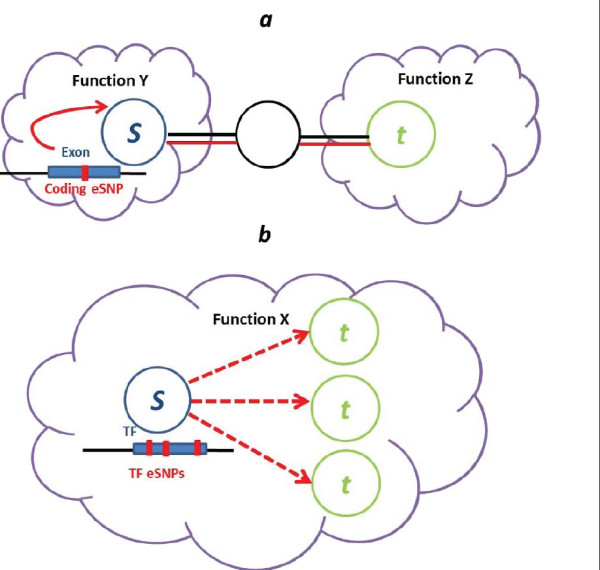
**Summary illustration - two suggested modes of *trans *regulation**. **(a) **Exon variation often acts by a local *cis *effect, delineating shorter paths of interacting proteins across functional clusters of the PPI network. **(b) **TF variation frequently acts via a modular regulation mechanism, with multiple targets that share a function with the TF source. (See Figure 3 legend for further details.) eSNP, expression single nucleotide polymorphisms.

Our main findings suggest two modes of *trans *regulation via genetic variation in exons and TFs. Exonic variation possibly acts through mild *cis *effects that alter the expression of the source gene and delineates shorter paths between functional clusters (Figure 4a), and exonic eSNP targets might play an important role in the PPI network as hubs. TF variation frequently acts via a modular regulation mechanism, with multiple targets that share a function with the TF source (Figure 4b).

Future studies could extend the approach presented here to investigate how genetic variation in different meaningful genomic locations (for example, enhancers, insulators, miRNAs) correlates with gene targets. Datasets that combine sequenced variants coupled with gene expression and phenotypic traits are limited in human, but available for other model organisms [50,51]. It would be insightful to combine this type of study with phenotypic data, to see how t*rans *association tracks with phenotypes. Specifically, applying our approach to samples under various conditions (for example, disease), could improve understanding of condition-specific regulatory processes [24]. Moreover, considering genetics-genomics data across different tissues along with a tissue-specific PPI network [52] could be telling regarding the underlying regulatory mechanisms characterizing these tissues.

## Methods

### Data details and processing

We analyzed a cohort of 50 Yoruban samples, for which genotypes of SNPs that are fully ascertained from sequencing data [29] along with RNA-sequencing data [30] are publicly available. Briefly, the raw dataset consists of 10,553,953 genotyped SNPs and expression measurements (quantile-quantile normalized values) of 18,147 genes with Ensembl gene ID across these 50 samples. Standard filters have been applied to the genetic data: minor allele frequency >0.05, SNP missingness rate <0.1 and individual missingness rate <0.1 [53]. After filtering, data for analysis consist of 50 samples with 7,206,056 SNPs.

### Association testing

For association analysis, we considered only SNPs that resided within candidate regulatory regions along the genome. For *trans *association, we tested for association between a SNP and every gene; we considered SNPs within the span of known exons and TFs (including introns) [54]. We tested for association using linear regression (Figure S2 in Additional file 1).

### Obtaining a random distribution of association test statistics

Examining the random distribution of association tests was helpful in evaluating the empirical significance of results. This was achieved by generating 100,000 random pairs of sources and targets for exonic and TF variation separately. We used a strict randomization process of edges switching. We picked a source gene from all sources in the real data; we then picked a target gene from all targets in the real data with a *P*-value cutoff of 10^-6^. When evaluating the number of targets per TF source, we created 1,000 sets of random TF source and gene target pairs; each set contained 370 such pairs corresponding to 370 TF source-target pairs at a *P*-value cutoff of 10^-6 ^in the real data.

### Identifying topological properties of source-target pairs projected on the PPI network

We used the PPI network provided by the Human Protein Reference Database [49]. The undirected network contains 9,671 nodes and 37,041 edges. For each node, we calculated its degree: the number of edges incident on the node. We defined a distance between every two nodes as the number of edges on the shortest path between them. All pair-wise shortest paths were determined using the Floyd-Warshall algorithm [55]. In cases where the network had more than one connected component, nodes from two different components were defined to have a distance of twice the maximal distance obtained within the components.

### Identifying topological trends across association P-values

For exons, we observed the emergence of true positive associations between *P-*values 10^-6 ^and 10^-7 ^(Figure S2a in Additional file 1). Therefore, we focused on *P*-values <10^-6 ^and sorted all source-target pairs according to the significance of their association signal. We considered each prefix of this list, that is, each subset of source-target pairs exceeding a particular threshold, for significance of association signal. For each such subset, we reported each one of the topological properties defined above averaged over the subset. We calculated Spearman's correlation coefficient between significance thresholds and each of these cumulative averages. In a similar process, we randomly chose an equal number of arbitrary source-target pairs on the PPI network. Adding these pairs one by one created a distribution of analogous cumulative averages for permuted pairs. We recorded the Spearman correlation coefficient for these 100,000 permuted distributions. We calculated the empirical *P*-value for the significance of the observed correlation coefficients by counting the number of times when permuted r > real r and divided this by the number of permutations.

### Expression analysis

We calculated all pairwise co-expression correlations for all gene pairs in the dataset using Spearman rank-correlation test, and therefore obtained the distribution of the correlation coefficient *r*. To determine whether the distribution of *r *between source-target pairs differed from its background distribution, we employed the Wilcoxon ranked-sum test.

### Enrichment of eSNPs for cis effects

We examined whether eSNPs that were associated with a target's expression level also affected expression levels of the corresponding source. We tested this by considering, for each source-target pair, the one eSNP most associated to the expression for the target. We tallied the source-target pairs for which this eSNP was also significantly associated (*P *<0.05) with the expression level of the source. Under the null, the number of such pairs is a random variable that is binomially distributed. Bin (n = number ofunique source genes, *P *=0.05).

### Unit and path annotation

We defined units of genes by considering a TF source and its gene targets. We examined shortest paths within the PPI network between eSNP exon source and its gene target. The enrichment of units and paths with gene subsets from the Gene Ontology [38], and KEGG [37] databases was calculated by Genatomy [36]. We reported only units or paths with annotations that had a significant FDR of 0.05 or better. The description of genes in units or paths is cited from the National Center for Biotechnology Information Gene database and GeneCards [56].

### Finding transcription factor source-target pairs in the experimental database

The ChIP Enrichment Analysis (ChEA) database [39] represents a collection of interactions describing the binding of transcription factors to DNA, collected from ChIP-X (ChIP-chip, ChIP-sequencing, ChIP-positron emission tomography and DNA adenine methyltransferase identification) experiments. For each TF source and target, we examined if they were present in ChEA. We repeated the same procedure for 100,000 permuted pairs of a random TF source and a random gene target. We then compared, using Fisher's exact test, the number of pairs in ChEA between real and permutation pairs, out of all pairs where the TF source was included in the database.

### Finding PPI network decomposition to clusters

The decomposition of the PPI network to clusters was computed by using the Louvain algorithm presented in [57]. This is a heuristic method that is based on modularity optimization. The method consists of two phases and partitions the network into clusters such that the number of edges between clusters is significantly less than expected by chance. The method provides a mathematical measure for modularity with network-size normalized values, ranging from 0 (low modularity) to 1 (maximum modularity). This method has been previously applied to various biological networks [58] and specifically to a PPI network [59].

### Significance of source and target residing in the same PPI cluster

For each exon and TF source-target pair, we recorded whether both source and target resided in the same PPI cluster. We repeated the same procedure with 100,000 permuted unique source-target pairs from nodes on the PPI network. We then compared the number of cluster co-occurrences between real data and permutations using the Fisher exact test.

### Comparing shortest paths annotation content

We recorded all genes along the shortest paths between exonic sources and targets, both in real and permuted data. We then looked for enrichment in this set of genes (at least 10 genes per category, FDR <0.05). We created sets of 1,000 permuted 55 shortest paths (from the 17,564 shortest paths in permutations) that followed the exact length distribution of the 55 real paths. For each one of the six categories that was not enriched in permutations, we performed two analyses: first, we counted how many genes from each category appeared in the real paths (with repetitions, that is if gene × from category Y appeared in two shortest paths we counted it twice); and second, we counted how many of the 55 paths had at least one gene from this category. We repeated the same procedures for the 1,000 permuted sets. For each category, we then counted how many of the 1,000 permutations achieved equal or greater numbers than seen for the real data (empirical *P*-value).

## Abbreviations

ChIP: chromatin immunoprecipitation; DHS: DNaseI hypersensitive site; eQTL: expression quantitative trait loci; eSNP: expression single nucleotide polymorphism; FDR: false discovery rate; GWAS: genome-wide association studies; Mb: megabase; PPI: protein-protein interaction; TF: transcription factor; *TCF7L2*: transcription factor 7-like 2; T-cell specific; *TLE4 *transducin-like enhancer of split 4; *MYC*: v-myc avian myelocytomatosis viral oncogene; catenin (cadherin-associated protein): beta 1, 88kDa (*CTNNB1*); *PIDD*: p53-induced death domain protein; *PLK3*: polo-like kinase 3; *EFEMP2*: Epidermal growth factor-containing fibulin-like extracellular matrix protein 2; *TP53*: tumor protein p53.

## Authors' contributions

AK conceived, designed and performed research, analyzed the data and drafted the manuscript. IP conceived and designed research and wrote the paper. Both authors read and approved the final manuscript.

## Supplementary Material

Additional file 1 **Supplementary text and figures**. Click here for file

Additional file 2 **Table S2**: Exon source with their corresponding eSNP targets, for each *P*-value smaller than 10^-6^, where a source-target pair on the PPI network was added, we recorded the differences between topological properties of random and real pairs using Wilcoxon rank sum test. The table includes for each *P*-value the number of unique pairs on the PPI network, the rank sum test *P*-values and the mean value for each one of the topological properties (distance and source and target degrees) for real and random pairs. Click here for file

Additional file 3 **Table S5**: For all TF and exonic source-target pairs we give the eSNP rs number, eSNP chromosome, eSNP location, source gene ID, target gene ID, target chromosome and association *P*-value. For eSNPs in TF, we indicate whether they are within an exon. Click here for file

Additional file 4 **Table S6**: Functional enrichment analysis of combined sets of exon sources, exon targets and TF targets (gene sets include only genes that map to an Entrez ID). Click here for file

Additional file 5 **Table S9**: TF units' functional enrichment (gene sets include only genes that map to an Entrez ID). Click here for file

Additional file 6 **Table S10**: Functional enrichment analysis of clusters in the PPI network (gene sets include only genes that map to an Entrez ID). Click here for file

Additional file 7 **Table S12**: Functional enrichment of exon paths, between source and target (gene sets include only genes that map to an Entrez ID). Click here for file

Additional file 8 **Table S13**: Enriched annotations (minimum 10 genes, FDR <0.05) of genes along real and permuted data shortest paths, and gene names for the six categories that were enriched in real shortest paths. Click here for file
