# Supplementary material for: Variants in exons and in transcription factors affect gene expression in trans
Source: Genome Biol. 2013 Jul 11;14(7):R71. doi: 10.1186/gb-2013-14-7-r71 (PMC4054683; doi:10.1186/gb-2013-14-7-r71)
Supplement: Additional file 1 — Supplementary text and figures. [file gb-2013-14-7-r71-S1.DOCX]

## **Variants in Exons and in Transcription Factors Affect Gene Expression *in Trans***

**Kreimer *et al.***

**Supplementary Data File (Additional file 1)**

**
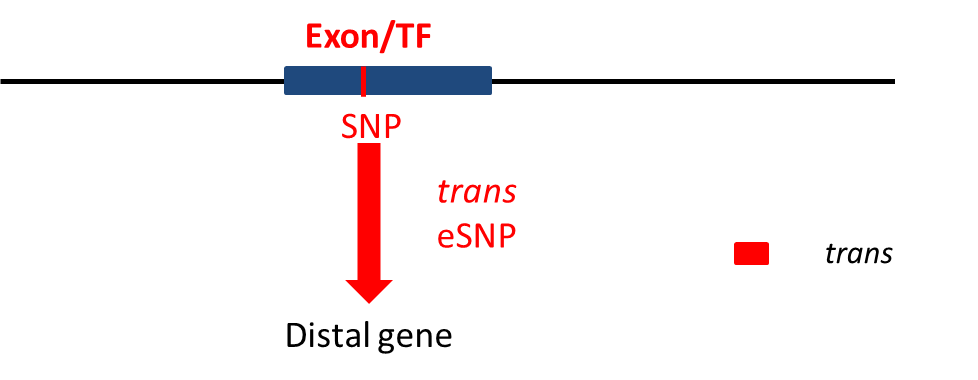
**

Figure S1: Illustrating the association testing between pairs of SNPs within known regulatory regions and genes: *trans* regulatory elements appear in red. If the regulatory element has an SNP within the boundaries of an exon or a TF then we check for nominal association (p < 0.05 denoted by a red edge) using linear regression between the minor allele count of the SNP and any gene.


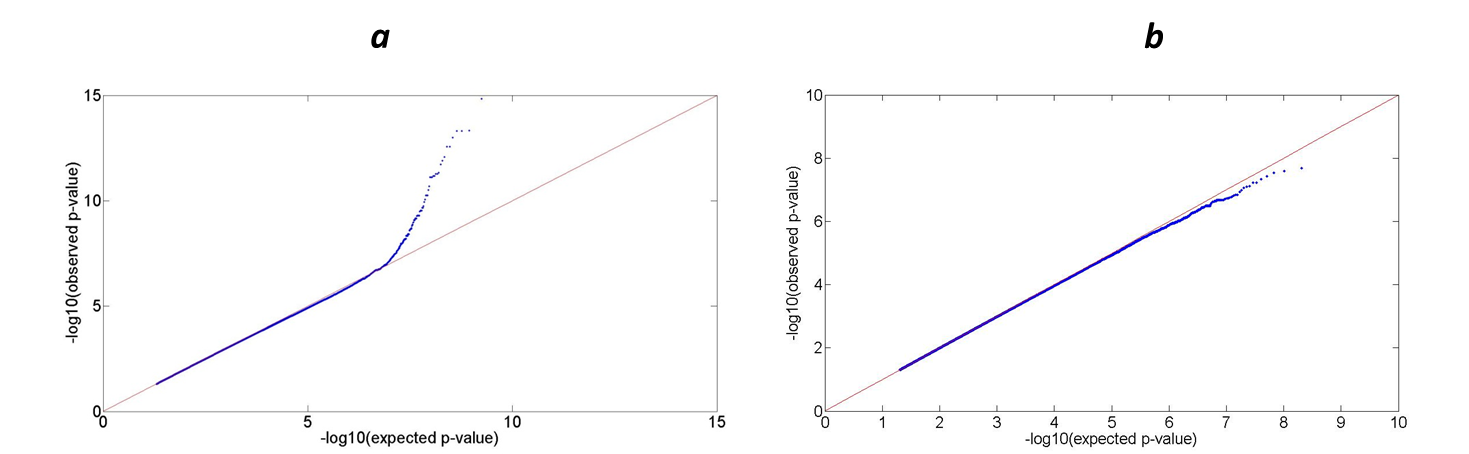


Figure S2: QQ plot for association pairs of SNPs within known regulatory regions and genes. (a) eSNPs in exons and (b) eSNPs in TFs. X-axis denotes -log 10 of the expected p-value. Y-axis denotes -log10 of the observed p-value. The red line denotes expectation by chance (Y=X).


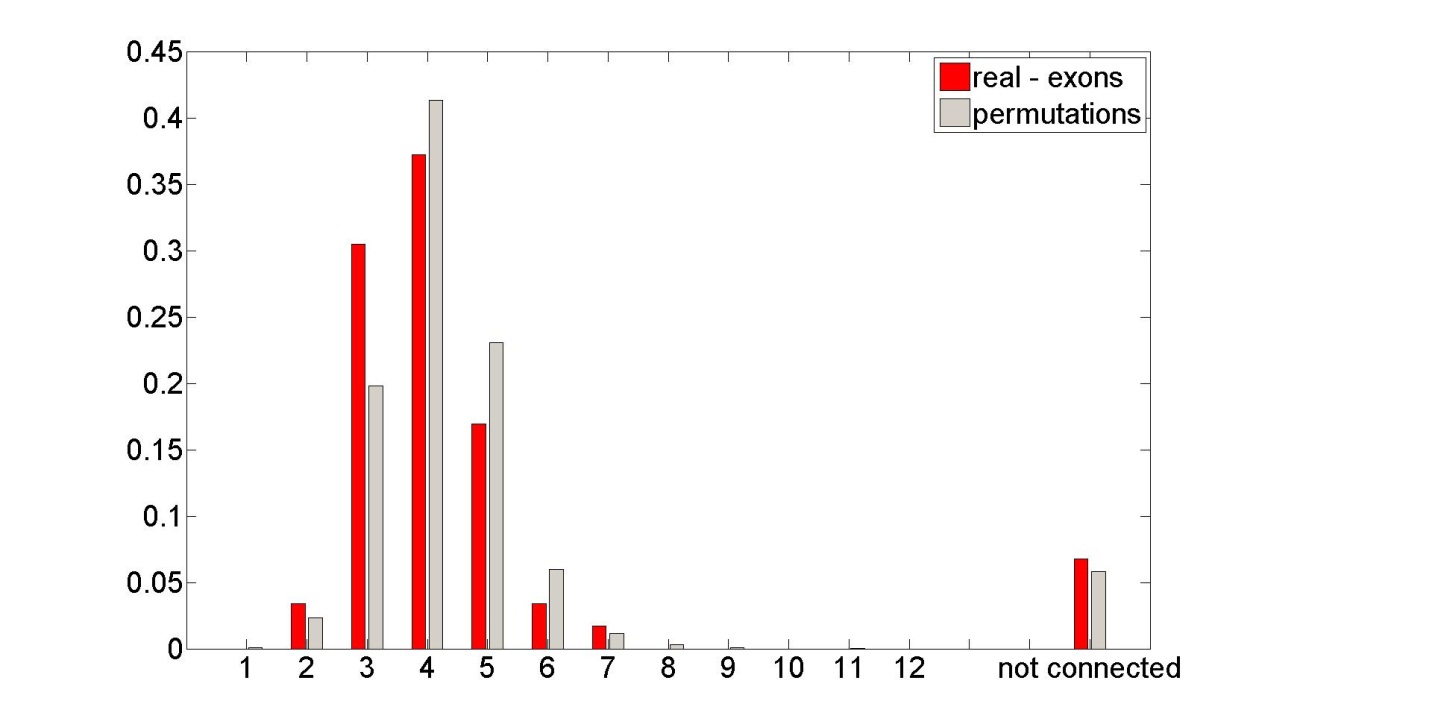


Figure S3: Histogram in percentage for the distances between pairs of exon source and target in real (red) and permuted (grey) data, for p-value=10^-6.463^.


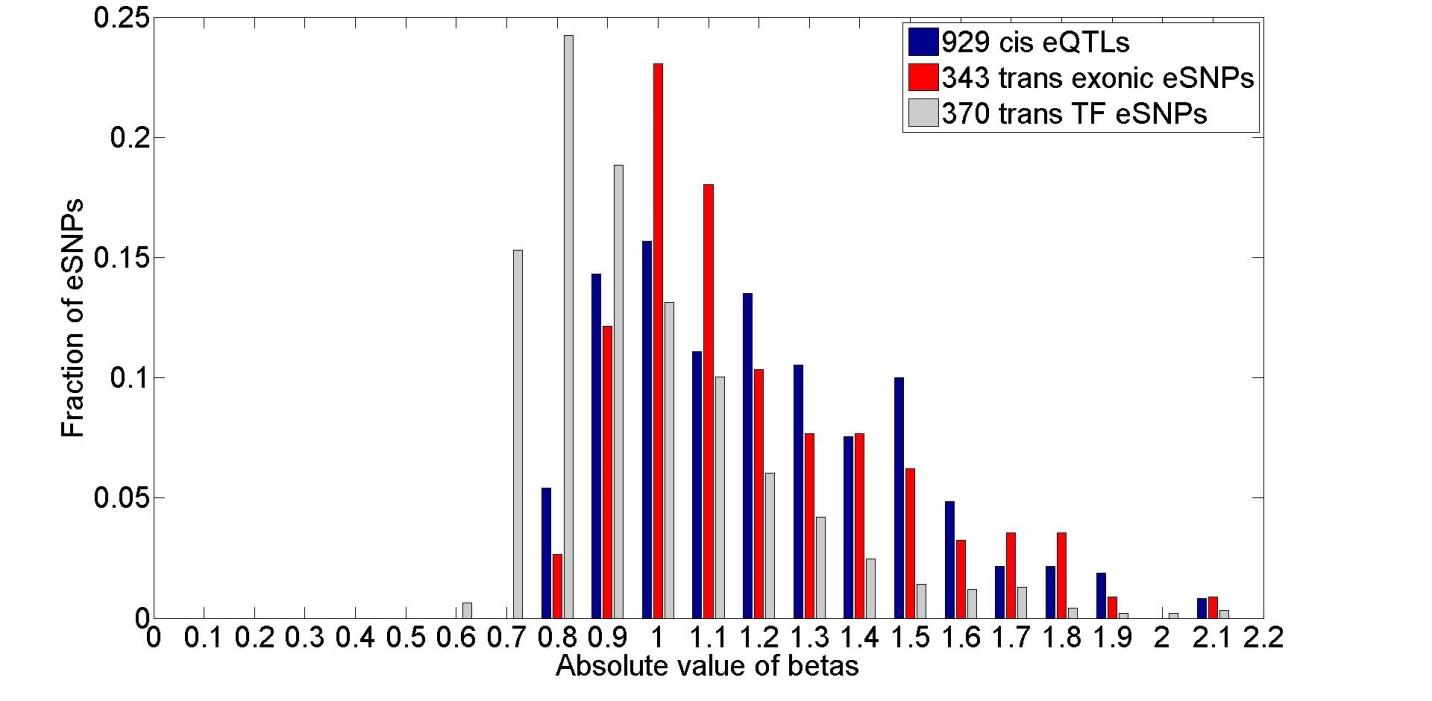


Figure S4: comparing effect sizes (absolute value of betas) between previously published 929 *cis* eQTLs and 343 and 370 exonic and TF *trans* eSNPs respectively.


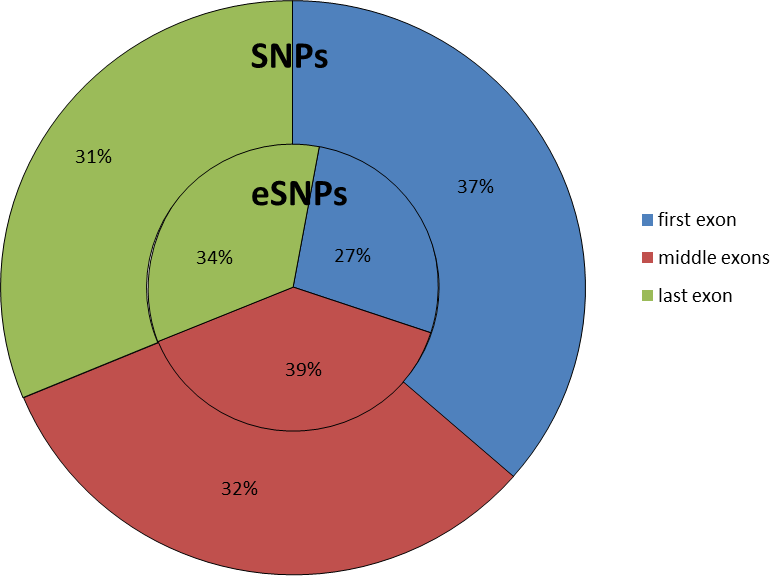


Figure S5: distribution of SNPs and *trans* eSNPs in exons.


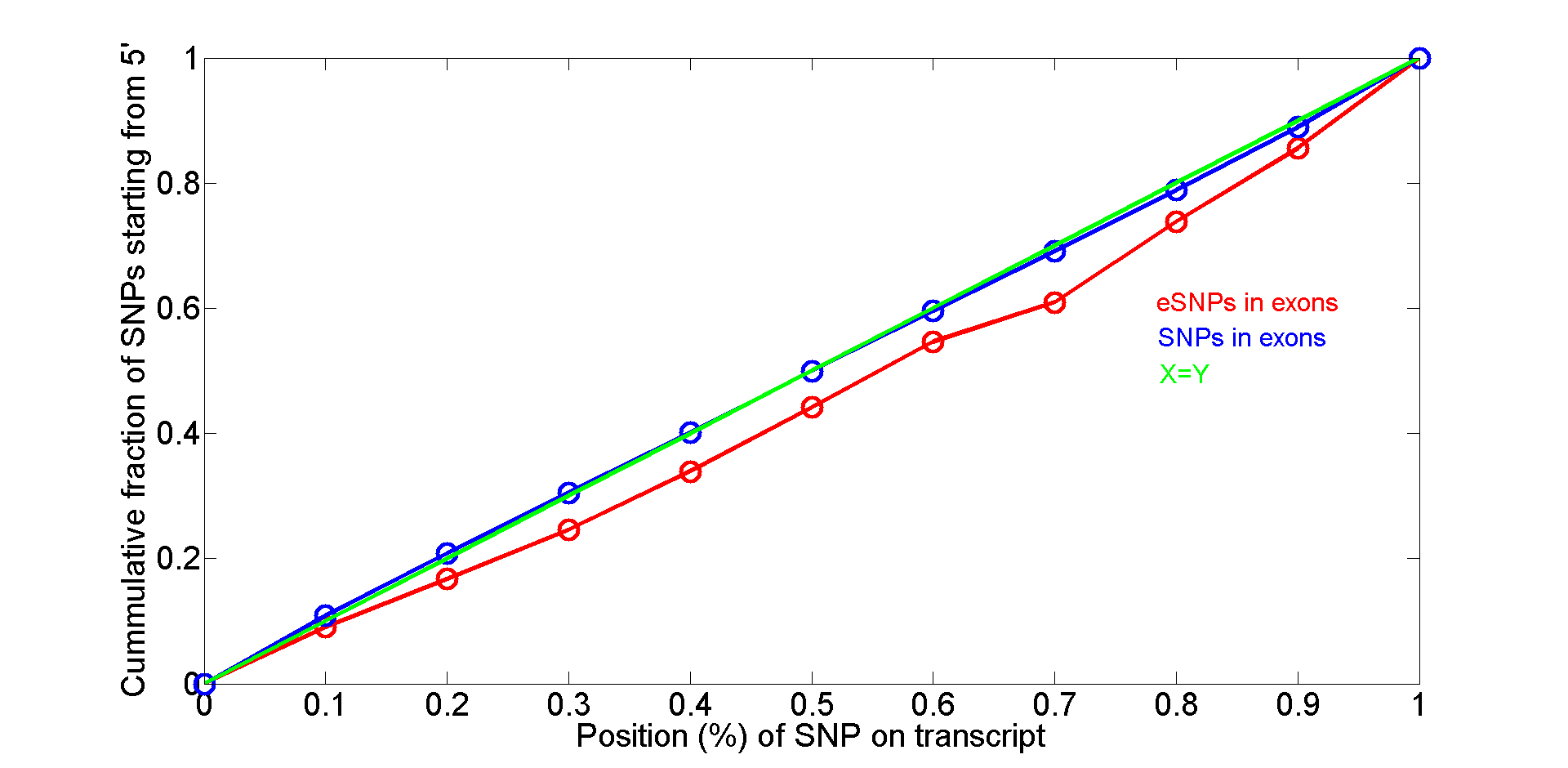


Figure S6: Cumulative fraction of the position of exonic eSNPs (red) and SNPs (blue) on the transcript (Wilcoxon rank sum test p-value between the position of exonic eSNPs and SNPs on transcript < 0.0058).


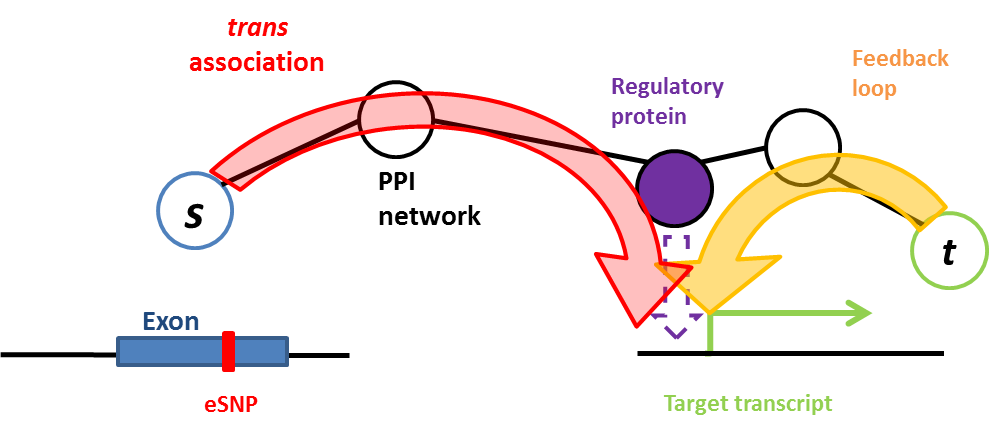


Figure S7: A path of interacting protein pairs (black circles and connectors) along the PPI network, from a source protein (blue) to the target transcript and protein (green), is consistent with concatenation of two pathways: the prefix of the path is consistent with a regulatory pathway (red), leading to some regulatory protein (purple node), that (directly or indirectly) affects expression of the target (purple arrow), thus being observed as a trans-eQTL signal. The suffix of the path may match a self feedback loop in reverse: from the target protein back to the same regulatory protein (orange arrow).

Table S1: Distribution of SNPs between regulatory elements and number of significant associations (*p*<0.05).

| Regulatory region | Number of SNPs | Number of eSNPs associations (*p* < 0.05) |
| --- | --- | --- |
| Exons | 95,957 | 88,267,318 |
| TF | 29,212 | 26,824,165 |

Table S2: in additional file 2.

Table S3: Distances between real exon source and target and between random pairs.

| Distance btw. pairs | Real – 59 pairs | Random – 18,675 pairs |
| --- | --- | --- |
| 2 | 2 | 431 |
| 3 | 18 | 3,701 |
| 4 | 22 | 7,723 |
| 5 | 10 | 4,308 |
| 6 | 2 | 1,118 |
| 7 | 1 | 212 |
| 28 (not connected) | 4 | 1,092 |

Table S4: Distribution of distances for source and target that are on the same chromosome for eSNPs in (a) exons (b) TFs.

(a)

| **Same chr** | **Distance** |
| --- | --- |
| 19 | 15002266 |
| 3 | 178621023 |
| 2 | 123847673 |
| 2 | 123623265 |
| 1 | 172680949 |
| 16 | 9843129 |
| 16 | 9841673 |
| 17 | 37168762 |
| 11 | 67193065 |
| 17 | 27955406 |
| 7 | 83684093 |
| 14 | 9357673 |
| 11 | 108362267 |
| 11 | 108387122 |
| 11 | 108386807 |
| 5 | 1278322 |
| 5 | 1336785 |
| 5 | 1367097 |
| 5 | 1359523 |
| 6 | 1437809 |
| 19 | 44903272 |
| 1 | 202997844 |
| 1 | 202973419 |
| 6 | 1468850 |
| 6 | 1443680 |

(b)

| **Same chr** | **Distance** |
| --- | --- |
| 2 | 64552602 |
| 1 | 204240596 |
| 1 | 204239155 |
| 1 | 204239711 |
| 2 | 36475062 |
| 1 | 39690140 |
| 1 | 127192199 |
| 1 | 127204055 |
| 1 | 39318770 |
| 1 | 228432802 |
| 1 | 35767071 |

Table S5: in additional file 3.

Table S6: in additional file 4.

Table S7: Units size distribution of TF source and their gene targets (units include only genes that map to an Entrez ID). We include the TF in the module.

| **Unit size** | **Number of units** |
| --- | --- |
| 2 | 35 |
| 3 | 16 |
| 4 | 8 |
| 5 | 2 |
| 6 | 2 |
| 8 | 1 |
| 10 | 2 |
| 11 | 1 |
| 17 | 1 |

Table S8: TF units sizes, TF source and gene targets (two or more) Entrez IDs.

| **Unit number** | **Unit size** | **TF source** | **Genes in the unit** |
| --- | --- | --- | --- |
| 1 | 3 | RUNX1 | CLN5, TCL1A |
| 2 | 3 | DMRT1 | EIF3H, GPATCH8 |
| 3 | 3 | GTF2F2 | GOLGB1, ATP2C1 |
| 4 | 3 | HSF2 | ABCC1, CCDC102A |
| 5 | 3 | NFIX | ORC2, CCDC91 |
| 6 | 3 | TCF4 | PNMT, PPHLN1 |
| 7 | 3 | TCF12 | 1-Dec, AZI2 |
| 8 | 3 | TFDP2 | SEMA6B, EXOC3L4 |
| 9 | 3 | MBTPS1 | SOCS2, PPAN |
| 10 | 3 | MTF2 | DNAH17, BTG3 |
| 11 | 3 | ATF7IP | UNC13A, RILP |
| 12 | 3 | PHTF2 | PPP1R16B, OTOGL |
| 13 | 3 | TFB2M | ZNHIT1, CAMKK1 |
| 14 | 3 | TCF7L1 | KCNQ4, SETDB2 |
| 15 | 3 | GABPB2 | PSMC6, ABHD5 |
| 16 | 3 | NFXL1 | HIST1H2BB, CHEK2 |
| 17 | 4 | ATF3 | NR3C1, SNORD26, INPP5E |
| 18 | 4 | NFYC | RBM5, CACNG4, STARD9 |
| 19 | 4 | GTF2A1L | CUL1, RNF24, LAMP3 |
| 20 | 4 | CNOT1 | PSMC2, PCGF6, THAP3 |
| 21 | 4 | WWTR1 | NFYB, FAM118B, FAM78B |
| 22 | 4 | TRERF1 | ADPRH, DNAJC5, NBPF23 |
| 23 | 4 | BACH2 | MYO7A, SNRPD1, SIRT7 |
| 24 | 4 | TFAP2D | PLA2G6, C8orf55, TMEM159 |
| 25 | 5 | BRF1 | WWC1, GRHL1, DDX54, DDX51 |
| 26 | 5 | AKNA | TCFL5, CCT5, SLC25A39, ALG8 |
| 27 | 6 | MITF | NEDD9, ARHGAP11A, TMEM51, MAGOHB, MIR589 |
| 28 | 6 | TCF7L2 | BOK, TLE4, NOP58, NAT10, TOR3A, |
| 29 | 8 | TEAD1 | ST3GAL3, DYNLT1, DBNL, GCNT4, PHF7, HNRNPA1L2, BTBD19 |
| 30 | 10 | STAT4 | ACO1, GNRHR, GYPC, PTRH2, MBOAT7, OBFC1, CORO6, UHMK1, PPTC7 |
| 31 | 10 | TCERG1L | SLC25A20, GATA2, ZNF3, LRPPRC, ABCA12, PCYOX1L, LBH, C16orf74, MIR1909 |
| 32 | 11 | MYT1L | APBB2, CDC25A, COL1A2, MMP7, SH3BP2, CWC27, NCAPH2, HNRPLL, ZMAT2, RPS26P6 |
| 33 | 17 | CAMTA1 | NFKBIE, QDPR, SKP1, CDK2AP1, TAOK2, GNB5, NECAP1, TMBIM4, PTRH2, VASH2, TMEM121, ZFP91, NHLRC2, H3F3C, C1orf190, SNORA81 |

Table S9: in additional file 5.

Table S10: in additional file 6.

Table S11: Exon paths lengths and genes in path from source to target.

| **Path number** | **Path length** | **Genes in path (from source to target)** |
| --- | --- | --- |
| 1 | 3 | HLA-C, LILRB1, HLA-A |
| 2 | 3 | HLA-C, LILRB1, HLA-G |
| 3 | 4 | CYBA, 4687, CSNK2A1, HNRNPC |
| 4 | 4 | DVL3, PPP2CA, TP53, DAXX |
| 5 | 4 | GATA3, ETS1, NR3C1, COPS6 |
| 6 | 4 | HLA-DQB1, CD4, PIK3R1, AKT1 |
| 7 | 4 | PITX2, KAT5, CDK1, AMPH |
| 8 | 4 | PTPRA, KCNA2, DLG1, PAX6 |
| 9 | 4 | RPS14, SMAD2, TSC2, MAPKAPK2 |
| 10 | 4 | TPI1, CFL1, ATXN1, KIAA2026 |
| 11 | 4 | SIP1, SNRPD2, EGFR, MET |
| 12 | 4 | MAP4K4, ITGB1, CRKL, EPOR |
| 13 | 4 | ERC1, YWHAG, LUC7L2, UNC119 |
| 14 | 4 | CLASP2, FEZ1, PRKCZ, GSK3A |
| 15 | 4 | GGA3, TSG101, NR3C1, SUMO4 |
| 16 | 4 | TES, ACTN1, GRIN2A, PTPN4 |
| 17 | 4 | PSMC3IP, NR3C1, PRKDC, EIF2S2 |
| 18 | 4 | PIDD, EFEMP2, TP53, PLK3 |
| 19 | 4 | MIF4GD, UBQLN4, IMPDH2, SUMO4 |
| 20 | 4 | STK11IP, SMAD4, MAPK13, MAPKAPK3 |
| 21 | 5 | BLK, BCL2, CDK2, PRKAR1A, C2orf88 |
| 22 | 5 | DYNC1H1, YWHAG, ARAF, TH1L, FRMD5 |
| 23 | 5 | STX2, STXBP1, PRKCA, TIAM1, MAPK8IP1 |
| 24 | 5 | RBPJ, HMGB1, C14orf1, NSF, NAPG |
| 25 | 5 | MUC4, ERBB2, PTPN18, GAB1, MAPK4 |
| 26 | 5 | MYO5A, DYNLL1, MTA1, CCNH, CDK2 |
| 27 | 5 | PIN1, CHPF, SMAD9, LNPEP, TNKS2 |
| 28 | 5 | RAB5A, TSC2, SMAD2, HDAC1, DNMT3B |
| 29 | 5 | RAC2, CUL1, SMAD3, GGA1, M6PR |
| 30 | 5 | ENC1, TGFBR1, FBXO34, SKP1, FBXL8 |
| 31 | 5 | MADD, PIDD, CRADD, LRIF1, RNF10 |
| 32 | 5 | NRXN1, SYT1, GOLM1, NIPSNAP3A, EPHX2 |
| 33 | 5 | PRDX6, RARA, COPS2, COPS6, WIPI2 |
| 34 | 5 | CAMKK2, CALM1, CAMK2G, GRIN2B, AP4M1 |
| 35 | 5 | MAST3, PTEN, CSNK2A2, SMURF1, NAA16 |
| 36 | 5 | PPIL2, HSP90AA1, WASL, SH3GL3, C11orf68 |
| 37 | 5 | PTRH2, AES, AR, CDC25A, PIM1 |
| 38 | 5 | DNAJB11, PTN, BCCIP, RAD51, DMC1 |
| 39 | 5 | KLHDC5, COIL, SMN1, BCL2, PPP3CA |
| 40 | 5 | HIF3A, HIF1A, CREBBP, MED25, MED15 |
| 41 | 5 | COL18A1, KDR, SRC, PRKACA, TPH1 |
| 42 | 5 | IQCG, BAG6, SMN1, KPNB1, UBR5 |
| 43 | 6 | CSF3, CSF3R, GRB2, EPHB6, SAT1, SAT2 |
| 44 | 6 | MUC2, PLEKHM1, EIF2S2, CSNK2A1, CDK1, NES |
| 45 | 6 | CLIP2, DYNLL1, TP53BP1, EP300, MYBL2, ZNF622 |
| 46 | 6 | PRPF4B, YWHAG, PRKCA, ITGB2, HP, C1RL |
| 47 | 6 | BRE, GFI1B, PSMA3, CDKN1A, RAB1A, ZNF593 |
| 48 | 6 | EDEM1, CANX, SMURF2, NEK6, CDK7, GTF2H2 |
| 49 | 6 | MAML1, CREBBP, EWSR1, RALYL, ZNF408, ZNF330 |
| 50 | 6 | SEC23B, SEC24D, LMO4, MERTK, BMPR2, PDZRN3 |
| 51 | 6 | CECR2, UXT, AR, RB1, TRIM27, FXYD6 |
| 52 | 6 | FBXO30, SMAD1, MAPK1, NEK2, NDC80, SPC25 |
| 53 | 7 | EIF4EBP2, EIF4E, PML, RELA, BRCA1, PSAP, CELSR1 |
| 54 | 7 | TNKS1BP1, TNKS, FNBP1, CDC42, WAS, CIB1, IFI6, |
| 55 | 8 | IRAK4, TRAF6, TRAF2, TCEA2, CENPT, PPCDC, DBI, TSPO |

Table S12: in additional file 7.

Table S13: in additional file 8.
